# Monitoring and simulating landscape changes: how do long-term changes in land use and long-term average climate affect regional biophysical conditions in southern Malawi?

**DOI:** 10.1007/s10661-023-11783-9

**Published:** 2023-09-26

**Authors:** C. Nkolokosa, Russell Stothard, Christopher M. Jones, Michelle Stanton, James Chirombo, Julie-Anne Akiko Tangena

**Affiliations:** 1 Sheffield Hallam University, Howard Street, Sheffield, S1 1WB UK; 2 Malawi-Liverpool-Wellcome Programme, Blantyre, Malawi; 3 Liverpool School of Tropical Medicine, Liverpool, L3 5QA UK

**Keywords:** Land use-land cover, Climate change, Machine learning, ANN, SVM, Malawi

## Abstract

We set out to reveal the effects of long-term changes in land use and long-term average climate on the regional biophysical environment in southern Malawi. Object-oriented supervised image classification was performed on Landsat 5 and 8 satellite images from 1990 to 2020 to identify and quantify past and present land use-land cover changes using a support vector machine classifier. Subsequently, using 2000 and 2010 land use-land cover in an artificial neural network, land use-land cover for 2020 driven by elevation, slope, precipitation and temperature, population density, poverty, distance to major roads, and distance to villages data was simulated. Between 1990 and 2020, area of land cover increased in built-up (209%), bare land (10%), and cropland (10%) and decreased in forest (30%), herbaceous (4%), shrubland (20%), and water area (20%). Overall, the findings reveal that southern Malawi is dominantly an agro-mosaic landscape shaped by the combined effects of urban and agricultural expansions and climate. The findings also suggest the need to enhance the machine learning algorithms to improve capacity for landscape modelling and, ultimately, prevention, preparedness, and response to environmental risks.

**Supplementary Information:**

The online version contains supplementary material available at 10.1007/s10661-023-11783-9.

## Introduction

Anthropogenic change is considered a pertinent environmental threat, and questions have been raised about the consequences of human-induced environmental change on the landscape, lives, and livelihoods across Malawi (Jørstad & Webersik, 2016; Kreft et al., 2016). Some of the questions to which there is a longing for answers are the following: What are the underlying mechanisms linking human activities and broad-scale landscape changes in Malawi? How do Malawi’s agricultural commercialization, macro-fiscal imbalances, and climate’s shock drive land use and habitat fragmentation in landscape mosaics? Firstly, it has been established that anthropogenic activities are causing significant changes in the extent of grassland, forest, marsh, and water habitats (Ministry of Natural Resources Energy and Environment, 2010). This is mostly linked to land conversion for crop production, over-harvesting of wetland vegetation, construction, brick making, sand extraction, charcoal burning, and water diversion for cultivation (Bone et al., 2017; Gondwe et al., 2021; Kpienbaareh et al., 2022; Mawenda et al., 2020; Ministry of Natural Resources Energy and Environment, 2010; Ngwira & Watanabe, 2019). Secondly, it is well known that climatic vicissitudes are devastating lives and livelihoods of Malawi, with southern Malawi being the most affected region (Jørstad & Webersik, 2016; Ministry of Natural Resources, Energy and Mining, 2016). For instance, southern Malawi was impacted by Tropical Cyclone Ana in January 2022 and subsequently Tropical Cyclone Gombe in March 2022 (Otto et al., 2022). Not surprisingly, both cyclones brought heavy rains and strong winds, causing floods, deaths, injuries, and infrastructure damage (UNICEF, 2022). However, the analysis of land use-land cover (LULC) to regularly monitor and assess biophysical landscape changes across the country remains lacking. Research to date have not yet determined how long-term changes in land use and long-term average climate relate to one another in driving and shaping the landscape in this geographical region. So far, the existing studies (e.g., Mungai et al., 2022; Mwale et al., 2014) have only modelled decadal land use-land cover change (LULCC) at the district level with no simulation on how the regional landscape will transition under climatic, topographic, and socioeconomic drivers. As a result, there is a paucity of direct knowledge on the combined effects of the land use and climate on land cover across the region.

Recently, however, in southern Malawi, the topic of environmental change is growing in importance in light of the increasing occurrence and intensity of climate hazards across the region (Kreft et al., 2016; Lee et al., 2021; Ministry of Natural Resources, Energy and Mining, 2016). The need exists for up-to-date information regarding the implications of the past and present land use and climate on the natural and man-made ecosystems such as croplands, settlements, wetlands, lakes, shrublands, and forests across the region. Providing LULCC insight will improve our understanding of the effects of land use and climate on the biophysical environment across mosaic landscapes, and what environmental changes are in store for such complex landscapes in the future. Ultimately, such knowledge will inform, for example, renewable energy and land policies and regulatory directions.

This study explores LULCC by integrating climatic and socioecological factors to better understand drivers and shapers of southern Malawi’s landscape. We first classified the LULC in 1990, 2000, 2010, and 2020 using a supervised machine learning algorithm, namely, support vector machine (SVM). This was followed by simulation of LULC in 2020 using the classified 2000 and 2010 maps, the drivers, and an artificial neural network (ANN) algorithm. The simulation of the LULC in 2020 was undertaken to determine whether the recent LULC in the study area is a product of the interactions between the climatic, topographic, and socioecological factors.

## Materials and Methods

### Study setting

**Fig. 1:**
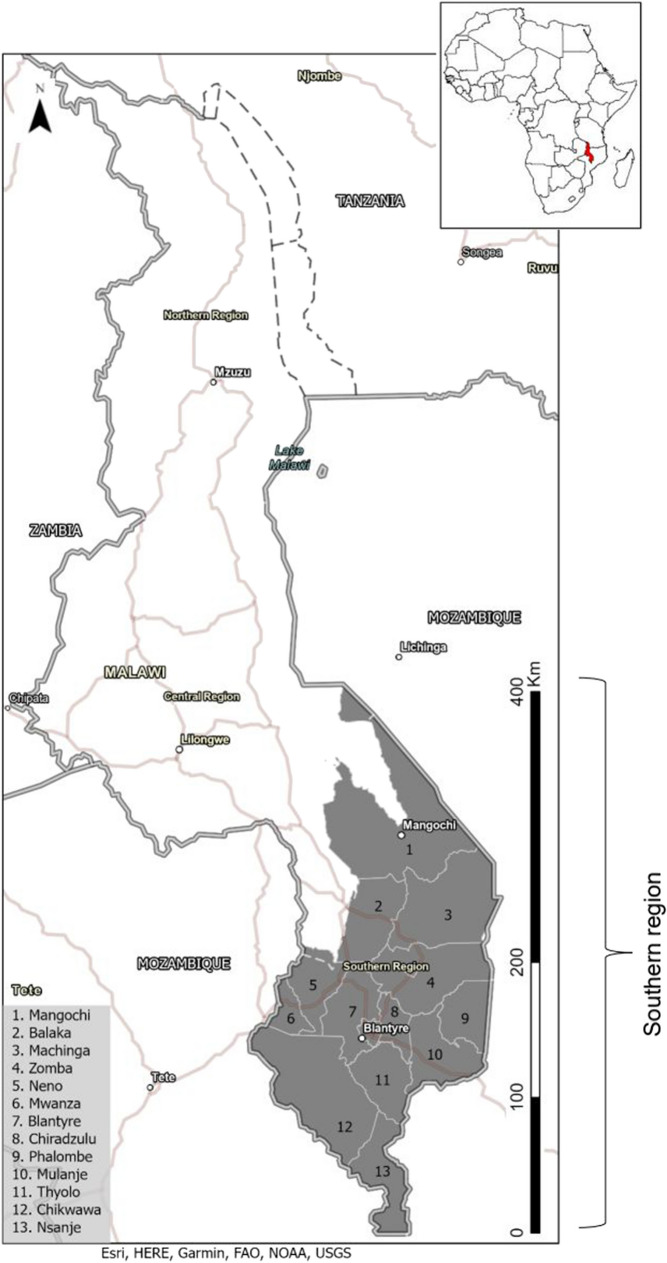
Southern Malawi: districts and study area. The inset map shows the location of Malawi in the context of Africa

The study area is southern Malawi (located between 14°25′ S and 16°55′ S latitude and 35°16′ E and 35°12′ E longitude) covering an area of approximately 32,644 km^2^ (Fig. 1). Southern Malawi is a low-income region with its population highly dependent on rainfed agriculture (Jørstad & Webersik, 2016), fishery resources (Jørstad & Webersik, 2016), and forest resources (Bone et al., 2017; Kalipeni, 1992; Ministry of Natural Resources, Energy and Mining, 2016; Ngwira & Watanabe, 2019). With most of the working population (85%) practicing rain-fed cultivation, the local population is heavily and directly dependent on the environment for their livelihoods, creating a perfect storm of deforestation, habitat loss and fragmentation, soil erosion, and many others (Ministry of Natural Resources, Energy and Environment, 2010).

Droughts and floods are the most severe and frequent climate hazards in the region—often causing loss of human life and livestock, crop destruction, property damage, and harm to natural resources (Ministry of Natural Resources, Energy and Mining, 2016). For example, the floods across the country alter landscapes: through erosional and sedimentation processes, and indirectly through forest loss as local communities expand their footprint (Bone et al., 2017). The dire consequences of floods are exemplified in the 2015 and 2019 Post Disaster Needs Assessment reports (FCFA, 2019). According to the reports, in 2015 and 2019, precipitation was four times higher above normal, resulting in heavy flooding in the southern region which caused human death and significant seasonal ecological changes (FCFA, 2019).

Anthropogenic activities and climatic shocks contribute to landscape changes in the region (da Silva Cruz et al., 2022; Gondwe et al., 2021; Joshua et al., 2016; Kalipeni, 1992; Kalipeni & Zulu, 2002; Mawenda et al., 2020). The seasonal changes in land use and worsening climate impacts across southern Malawi make this region an excellent case study of how climate changes and anthropogenic activities affect the landscape patterns.

### Datasets

This study uses the Landsat 5 Thematic Mapper (TM) and 8 Operational Land Imager (OLI) sensors (Poursanidis et al., 2015). The Landsat OLI and TM have been chosen for five reasons: (1) the imagery dataset covering the study area has a high temporal resolution—spanning from as early as the 1990s up to the 2020s, (2) they provide medium spatial resolutions from 15 to 30 m/pixel and high spectral resolution from 7 to 11 bands, (3) evaluation of the quality of the data showed that adequate imagery with low cloud cover (less than 5%) is available for the study area, (4) the imagery is open access, and (5) they provide adequate coverage of the whole study area, unlike Landsat 7 Enhanced Thematic Mapper images which have scan gaps, leading to missing scenes. The satellite images were obtained from the open-access Google Earth Engine Explorer (http://www.code.earthengine.google.com), a web-based computing platform for the Earth Engine JavaScript API. From this platform, yearly median (January–December) Landsat composite images acquired in 1990, 2000, 2010, and 2020 with less than 5% cloud, covering the entire southern Malawi, were downloaded and utilized for the supervised classification (see Supplementary Material Appendix A). Here, we used annual composites to minimize stochastic landscape changes that occur over shorter time periods and allow comparison of three decades using a common temporal unit of analysis, in this case, a year.

Table 1 presents a summary of the climatic and socio-economic variables used in the subsequent supervised classification. Administrative boundary, major roads (highway and primary road), and village shapefiles for the region were obtained from the Malawi Spatial Data Platform (MASDAP, http://www.masdap.mw/). Historic climate data, specifically average temperature and precipitation for the years 1970–2000, were downloaded from WorldClim (http://www.worldclim.org/). Gridded population density data (2000–2020) having 1 km spatial resolution were downloaded from WorldPop (http://www.worldpop.org/datacatalog/). The Digital Elevation Model (DEM) data, 30 m spatial resolution, was downloaded from the RCMRD Open Data online portal (http://www.opendata.rcmrd.org/datasets/malawi-srtm-dem-30meters/explore). The slope was derived from the DEM using the terrain analysis function within QGIS 3.28 (see http://www.docs.qgis.org/rasters/terrain/analysis). Poverty level raster data were downloaded from the MASDAP. The metrics used to measure the poverty levels range from income/expenditure, assets, and access to health, sanitation, and education services. The datasets were all projected to WGS 84 UTM Zone 36 South and resampled to 1 km × 1 km spatial resolution to match the geometries, thereby, precluding a random pixel not representing the same size of LULC in the other raster layers.

**Table 1:** Remotely sensed and GIS datasets used for the LULC mapping and simulation

Data	Spatial resolution	Source	Year(s)	File format
Landsat 5 TM	30 m	Google Earth Engine	1990, 2000 and 2010	GeoTIFF
Landsat 8 OLI	30 m, 15 m	Google Earth Engine	2020	GeoTIFF
Administrative boundary	-	HDEX	2017	Vector
Precipitation	~1 km	WorldClim	1970-2000	GeoTIFF
Temperature	~1 km	WorldClim	1970-2000	GeoTIFF
Population density	1 km	WorldPop	2000-2020	GeoTIFF
Elevation	30 m	RCMRD	2018	GeoTIFF
Slope	30 m	Calculated from DEM	2018	GeoTIFF
Poverty	100 m	MASDAP	2019	GeoTIFF
Major roads	-	MASDAP	2014	Vector
Villages	-	MASDAP	2013	Vector

For this study, major roads and village location data in vector format were used to create raster layers of Euclidean distance to major roads and villages, respectively. We then used the proximity variables together with a suite of other climatic, topographic, and socioeconomic drivers—temperature, precipitation, elevation, slope, population density, and poverty—to simulate recent LULCC across the study area (Supplementary material Appendix B, C and D).

### Data normalization

Recognizing that in machine learning, using raw input data tends to cause reduced accuracy and speed of ANN training, and the explanatory variables were normalized (Ostad-Ali-Askari et al., 2017). In an ANN, the distribution of the data is not assumed; hence, normalization becomes useful when the input data has varying scales. Additionally, since ANNs incorporate weights, ensuring that all the predictor variables have a common numerical range is therefore essential (Omrani et al., 2012). It is for these reasons that in the present study, all the explanatory variables were normalized to a common numerical range using Eq. (1) in ArcGIS Raster Calculator, resulting in values between 0 and 1.

1 $${X}_{\textrm{normalized}}=\frac{\left(X-{X}_{\textrm{minimum}}\right)}{\left({X}_{\textrm{maximum}}-{X}_{\textrm{minimum}}\right)}$$

where *X*, *X*_normalized_, *X*_minimum_, and *X*_maximum_ represent input variable values, the normalized value, and the possible minimum and maximum values, respectively (Ostad-Ali-Askari et al., 2017).

### Training sample collection

We used a seven-LULC classification schema: built-up, forest, herbaceous, bare land, water, cropland, and shrubland (Table 2). This classification schema was derived from the 2013 Atlas of Malawi Land Cover and Land Cover Change (FAO, 2020) and the LULC categories that could be identified from the segmented Landsat TM and OLI images (Supplementary material Appendix A), alongside the first author’s knowledge of the local landscape. Geographically uniformly distributed samples were collected across the study area from the segmented Landsat images using ArcGIS Pro’s Image Classification Wizard.

**Table 2:** LULC classification schema

ID	Landcover	Description
1	Built-up	Developed areas and artificial surfaces, such as settlement (urban and rural), industrial, and roads. Features indicating building footprint were assigned to this category.
2	Forest	Woodland and broadleaved deciduous trees in a stand.
3	Herbaceous	Vegetation having no persistent woody plants, such as grasses, reeds, and sugarcane.
4	Bareland	Areas with exposed surfaces such as bare rock, dried up rivers, and lakeshore
5	Water	Flowing and standing waterbodies
6	Cropland	Areas used for cultivating rain-fed crops, including ploughed fields
7	Shrub land	Areas characterised by low shrubs and widely dispersed trees

### Supervised image classification

To identify and quantify LULCC, this study adapts the methods and best practices of LULCC analysis and mapping used by the Food and Agriculture Organization (FAO); see FAO (2020) and Lam (2008). The methods involve (1) the use of supervised machine learning algorithms to process and segment imagery and (2) interpreting and validating outputs using existing land cover atlases and local knowledge. Training samples were collected by selecting segments—group of pixels characterized by a uniform color representing a class—from the segmented images using the Training Sample Manager Segment Picker in ArcGIS Pro 3.0.0. For each LULC class, we collected a minimum of 25 segments. Using the training samples, supervised classification was subsequently performed on the segmented Landsat ETM and TM imagery. Of note, a near-infrared (NIR) or color infrared composite (NIR, red and green Landsat band combination) was used for the training sample collection and supervised classification.

Here, an SVM algorithm was used to classify the LULC between 1990 and 2020. The SVM classifier has been chosen here because of its superior performance when compared to a parametric classifier, such as the maximum likelihood classifier, as demonstrated by Abdi (2020), Bahari et al. (2014), Candade et al. (2004), and Rokni Deilmai et al. (2014). The SVM classifier performs well because it is less susceptible to noise, related spectral bands, and inconsistent number of training samples within each LULC category (Pal & Mather, 2005; Rokni Deilmai et al., 2014). As a non-parametric classifier, the SVM classifier does not require normally distributed samples and thereby can classify the data nonlinearly. In principle, it classifies the images by determining the boundaries in feature space and allocates the pixel of land cover class to a single class (Bahari et al., 2014). A detailed mathematical description of SVM is given in Cortes and Vapnik (1995) and Candade et al. (2004).

### Annual rate of change

To better understand temporal change in urbanization, agricultural expansion, water body area, and deforestation, annual rate of change was computed as follows:

2 $$\textrm{R}=\left\{\frac{1}{t_2-{t}_1}\right\}\ast \left\{\ln \frac{A_2}{A_1}\right\}\ast 100$$

where *R* is the rate of change per year in percentage, *A*_1_ and *A*_2_ are the area in square kilometre at the beginning and end of the analysis period, and *t*_1_ and *t*_2_ correspond to the time in years from start to finish (Mawenda et al., 2020).

### LULC simulation

We employed the ANN-multi layer perceptron (MLP) model using the Modules for Land Use Change Simulations (MOLUSCE) in QGIS 2.18 to simulate LULC in 2020, based on LULCC between 2000 and 2010. The ANN-MLP model is a non-linear classifier and hence offers a more realistic way of simulating complex LULC transitions driven by a set of complex factors (Gharaibeh et al., 2020). To detect land transition and simulate LULCC scenarios, the model computes the functional relationship between the inputs, in this case, the LULC classes and the explanatory variables (Charif et al., n.d.). The mathematical function of the ANN-MLP model is given by Eq. (3):

3 $${y}_k=\Psi \left(\sum_{j=1}^p{v}_{jk}\Phi \left(\sum_{i=1}^q{\omega}_{ij}{x}_i+{\omega}_{0j}\right){v}_{0k}\right)$$

where *y*_*k*_ is the output (in this case, built-up, forest, herbaceous, bare land, water, cropland, and shrubland) expressed as a function of the input *x*_1_, *x*_2_, …, *x*_*q*_ (in this case, LULC and the explanatory variables). *ω*_*ij*_ and *v*_*jk*_ are weights assigned to the connections between the input layer and the hidden layer, and between the hidden layer and the output layer, respectively, *ω*_0*j*_ and *v*_0*k*_ are biases (or threshold values in the activation of a unit). Φ is an activation function, applied to the weighted sum of the output of the preceding layer (in this case, the input layer). Ψ is also an activation function applied, by each output unit, to the weighted sum of the activations of the hidden layer (Omrani et al., 2012).

In summary, the input layers receive the input data containing LULC classes and values of the explanatory variables described above and pass these to the hidden layer or artificial neurons. In the hidden layer of neurons, each neuron relates to each neuron of the next hidden layer by weighted input signals. The weights are summed up by the neurons and propagated to the output layer through nonlinear and linear transfer functions. To learn the weights, the ANN-MLP model finds the values that minimize the error by trying several different numbers as the weights.

### Accuracy assessment

Given that LULC classification and simulation is not a consistent process, the results from the supervised LULC classification and prediction were validated, based on methods adapted from FAO (2020), Appiah et al. (2015), and Mukherjee et al. (2009). This includes calculating the kappa coefficient and “ground truthing” in Google Earth. High-spatial resolution Google Earth imageries (1989–2020) were used to verify the classified maps. The kappa measures the goodness-of-fit between the actual scenario and predicted scenario (Appiah et al., 2015). The kappa values <0 signify no agreement, 0–0.2 as slight agreement, 0.2–0.41 as fair, 0.41–0.60 as moderate, 0.60–0.80 as substantial, and 0.81–1.0 as almost perfect agreement (Appiah et al., 2015; Landis & Koch, 1977). Mathematically, kappa is expressed as

4 $$\kappa =\frac{N\sum_{i=1}^r{X}_{ii}-\sum_{i=1}^r\left({x}_{i+}\right)\left({x}_{+i}\right)}{N^2-\sum_{i=1}^r\left({x}_{i+}\right)\left({x}_{+i}\right)}$$

where *r* is the number of rows in the matrix, *X*_*ii*_ is the number of observations in row *i* and column *i* (the diagonal elements), *x* + 1 and *x*_*i*_+ are the marginal totals of row *r* and column *i*, respectively, and *N* is the number of observations (Mukherjee et al., 2009).

Additionally, we used the percentage of correctness metric and learning curve graph outputted by the MOLUSCE to evaluate the performance of the simulation model. The MOLUSCE determines the latter by calculating false predictions in the simulated map using a two-map comparison approach (Gharaibeh et al., 2020).

## Results

### Temporal patterns in LULCC from 1990 to 2020

**Fig. 2:**
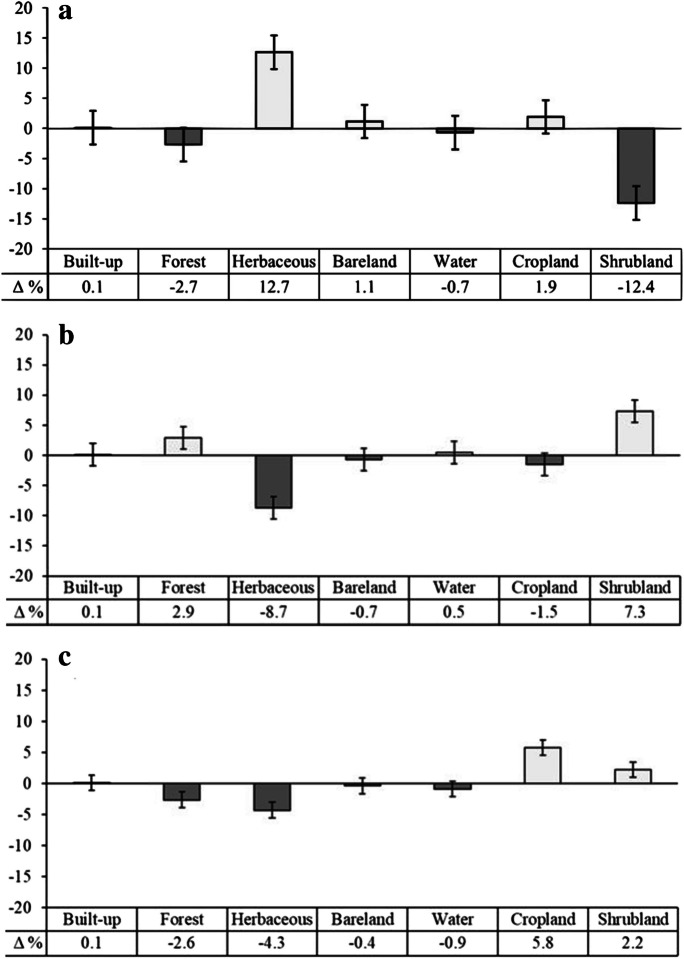
Percentage change in LULC between 1990 and 2000 (**a**), 2000 and 2010 (**b**), and 2010 and 2020 (**c**)

The results from the LULC classification indicate that between 1990 and 2000, herbaceous area, cropland, bare land, and built-up area increased by 12.7%, 1.9%, 1.1%, and 0.1%, respectively (Fig. 2a). In comparison, shrubland, forest land, and water area decreased by 12.4%, 2.7%, and 0.7%, respectively (Fig. 2a). Between 2000 and 2010, shrubland, forest, waterbodies, and built-up area increased by 7.3%, 2.9%, 0.5%, and 0.1%, respectively, while herbaceous area, cropland, and bare land shrunk by 8.7%, 1.5%, and 0.7%, respectively (Fig. 2b). Between 2010 and 2020, cropland, shrubland, and built-up increased by 5.8%, 2.2%, and 0.1%, respectively (Fig. 2c). In comparison, forest, herbaceous, bare land, and water decreased by 2.6%, 4.3%, 0.4%, and 0.9%, respectively (Fig. 2c). Thus, over the 30-year period, there was an increase in built-up, bare land, and cropland and a decrease in forest, herbaceous, water, and shrubland. During the same period, southern Malawi was dominantly an agro-mosaic landscape.

### Spatial patterns of LULC from 1990 to 2020

**Fig. 3:**
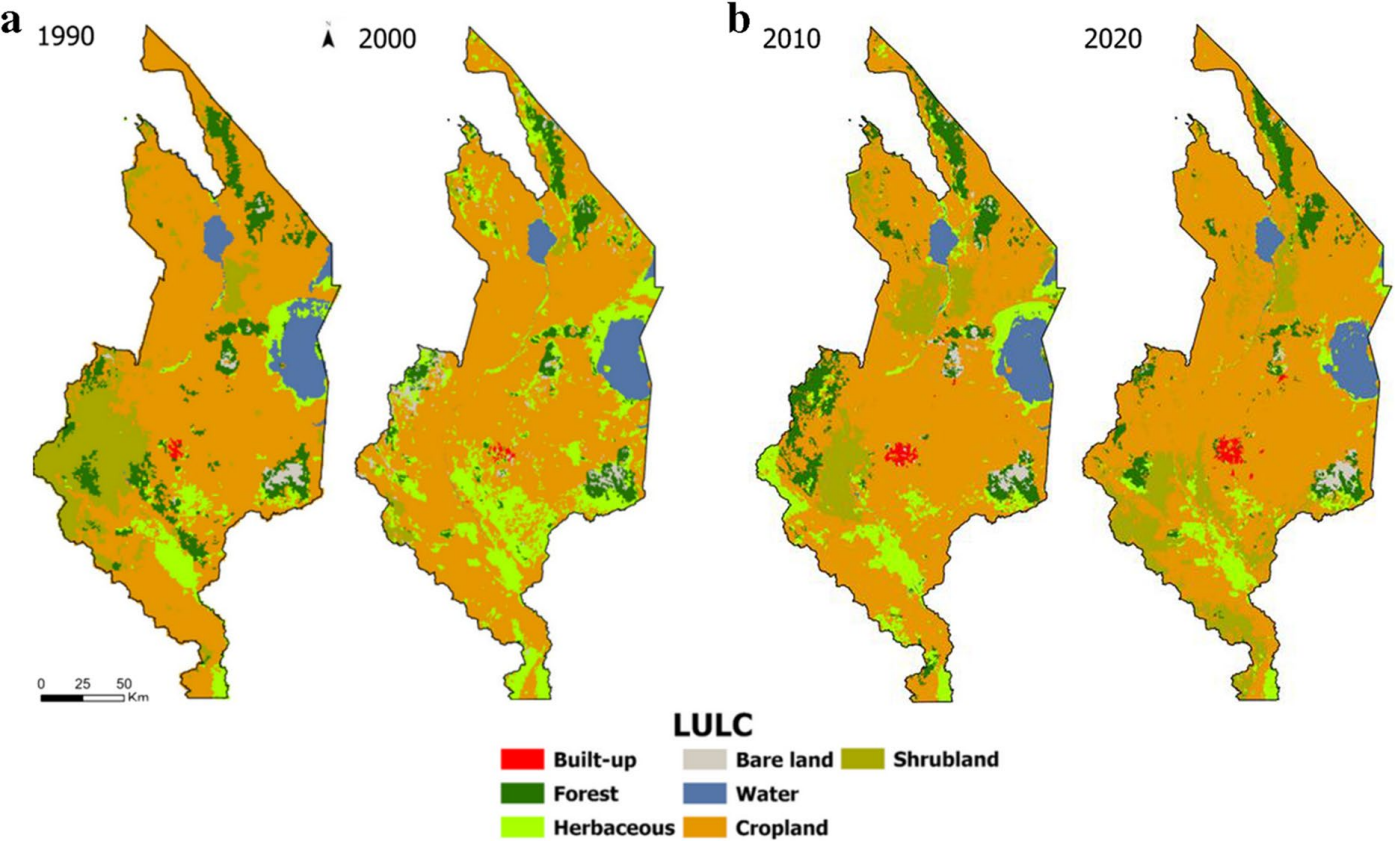
Spatial patterns in LULC distribution in 1990, 2000, 2010, and 2020 in southern Malawi

**Fig. 4:**
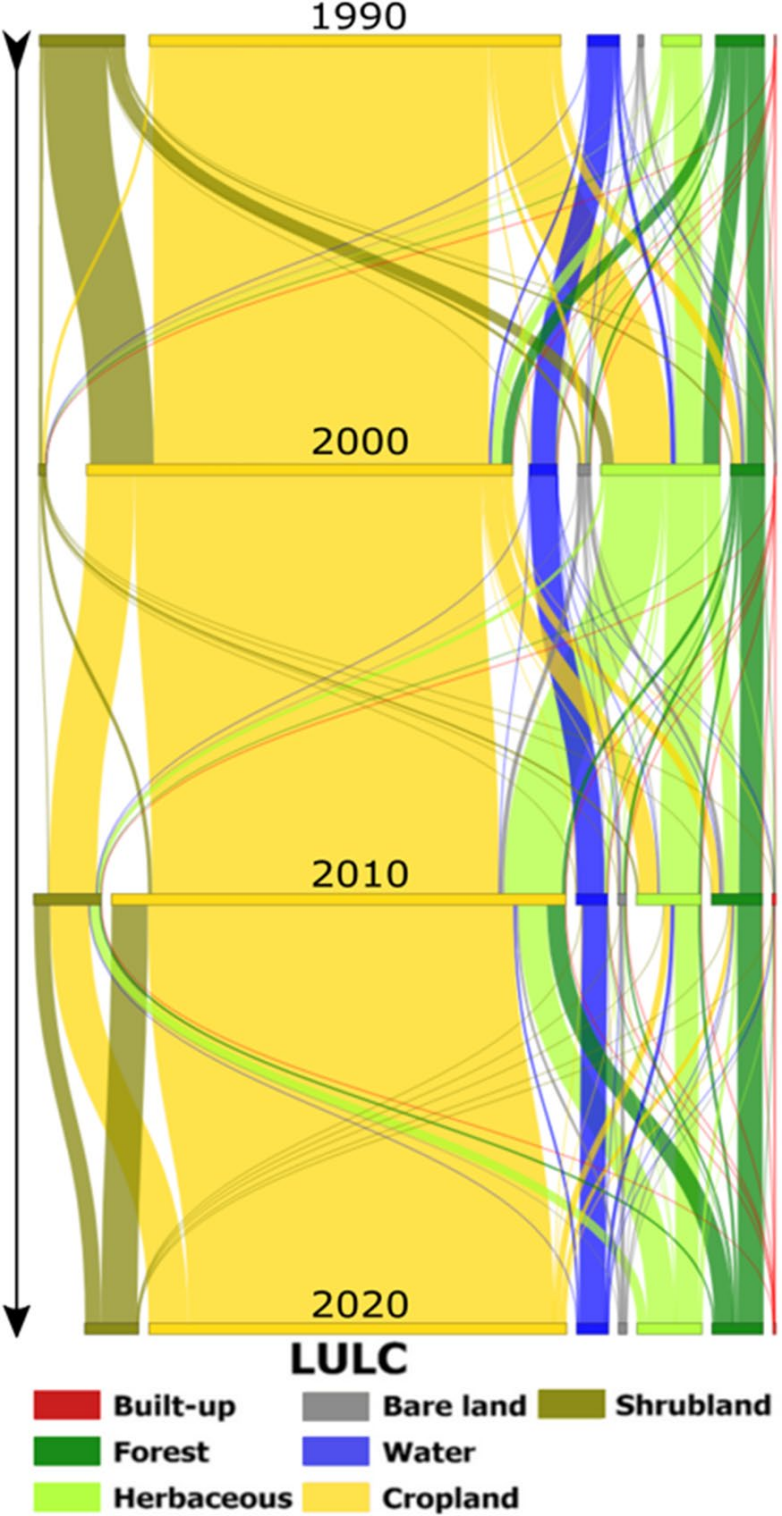
Dynamics of LUCC from 1990 to 2022 across southern Malawi. Note that the connection width (thin or thick) is proportional to the LULCC (small or large)

Figures 3 and 4 are clear demonstrations of the spatiotemporal distribution of built-up, forest, herbaceous, bare land, water, cropland, and shrubland in southern Malawi over 30 years. What is striking is cropland dominance and the perturbations in forest, herbaceous, and shrubland areas. Losses in vegetation cover were higher during the 1990–2010 period, evidently driven by cropland expansion. In the 2010–2020 period, vegetation gains are apparent. Here, bare land and built-up areas are lesser. Table 3 shows the estimated quantities of the LULC categories over the four time periods. Overall, net gains in built-up (115 km^2^), bare land (28 km^2^), and cropland (2239 km^2^) and net losses in forest (−763 km^2^), herbaceous (−84 km^2^), water (−344 km^2^), and shrubland (−897 km^2^) are evident.

**Table 3:** Area of LULC in 1990, 2000, 2010, and 2010

LULC	1990 (sq.km)	2000 (sq.km)	2010 (sq.km)	2020 (sq.km)
Built-up	55	87	134	170
Forest	2560	1680	2655	1797
Herbaceous	2073	6191	3398	1989
Bare land	287	661	435	315
Water	1701	1472	1651	1357
Cropland	21538	22116	21856	23777
Shrubland	4437	388	2810	3540

### SVM model performance

Table 4 below shows the kappa coefficient for the 1990, 2000, 2010, and 2020 classified maps. Overall, the kappa values of ≥ 0.85 indicate that the goodness of fit between the ground truth data and the classified maps is almost in perfect agreement. This means that the accuracy of the LULC classification for 1990, 2000, 2010, and 2020 Landsat imagery by the SVM classifier was up to standard.

**Table 4:** LULC classification accuracy for 1990, 2000, 2010, and 2020 images by the SVM classifier

Satellite imagery	Kappa value (𝜅)	Agreement level
1990 Landsat 5 TM	0.91	Almost perfect
2000 Landsat 5 TM	0.85	Strong
2010 Landsat 5 TM	0.89	Strong
2020 Landsat 8 OLI	0.94	Almost perfect

### LULCC simulation for 2020

**Fig. 5:**
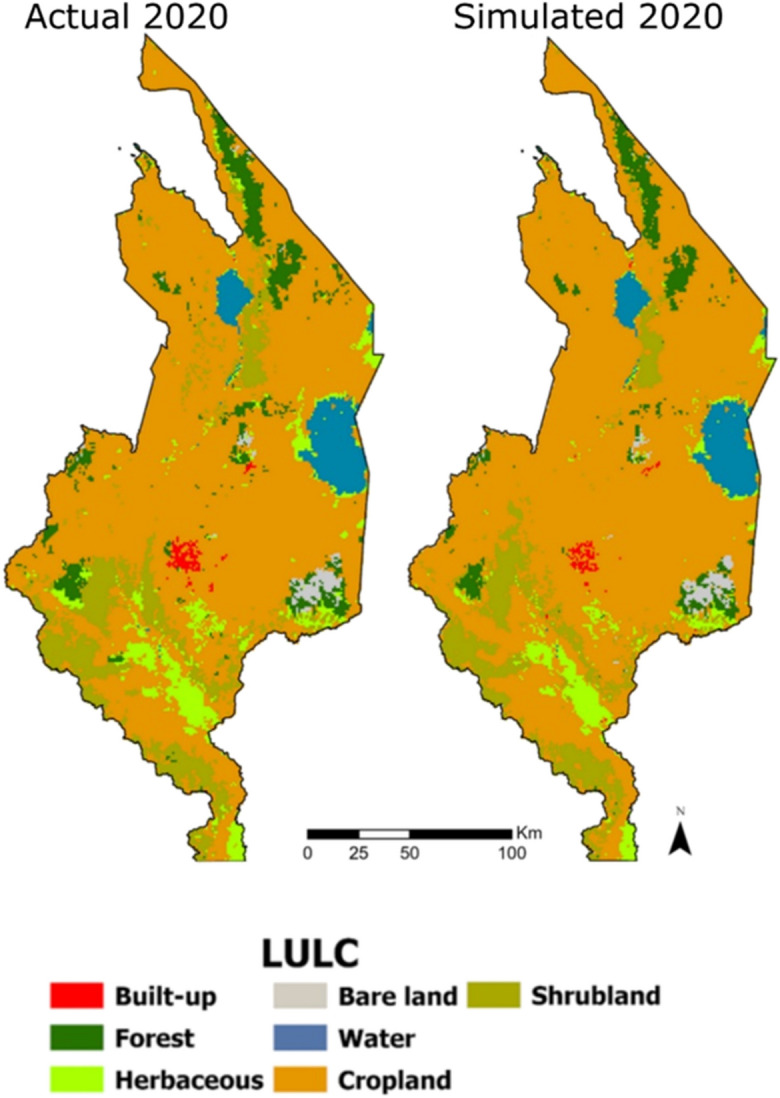
Comparison of the observed or classified map with the best predicted map

The prediction of LULC in 2020 using a fine-tuned CA ANN-MLP model produced a kappa coefficient of 0.73 (73%) and a percentage of correctness of 85.2% (Table 5). This means that the simulated map showed good agreement with the reference map (actual LULC for 2020). Overall, this result shows that the climate, topographic, and socioeconomic predictor variables provided acceptable LULC simulation results. This is reflected visually in Fig. 5 and quantitatively in Table 5. A comparison of observed (actual) and simulated LULC maps for 2020 indicates almost similar spatial patterns in LULC across the study area.

**Table 5:** Performance of the trained neural network

Learning rate	Maximum iterations	Hidden layers	Kappa overall (*𝜅*)	% of correctness
0.001	2000	500	0.73	85.2

Table 6 shows the LULC area in the observed and simulated maps. From the table, in the simulated LULC, the area of built-up (131 km^2^), forest (1403 km^2^), herbaceous (1462 km^2^), and water (1343 km^2^) was slightly lower when compared to the actual LULC area. On the other hand, cropland (25101 km^2^) and bare land (332 km^2^) were slightly overestimated.

**Table 6:** Comparison of observed and simulated LULC

LULC	Observed 2020 (sq.km)	Simulated 2020 (sq.km)	Actual 2020 (%)	Simulated 2020 (%)
Built-up	170	131	0.5	0.4
Forest	1797	1403	5.5	4.3
Herbaceous	1989	1462	6.0	4.4
Bare land	315	332	0.9	1.0
Water	1357	1343	4.1	4.0
Cropland	23777	25101	72.2	76.2
Shrubland	3540	3173	10.7	9.6

### CA ANN-MLP model performance

**Fig. 6:**
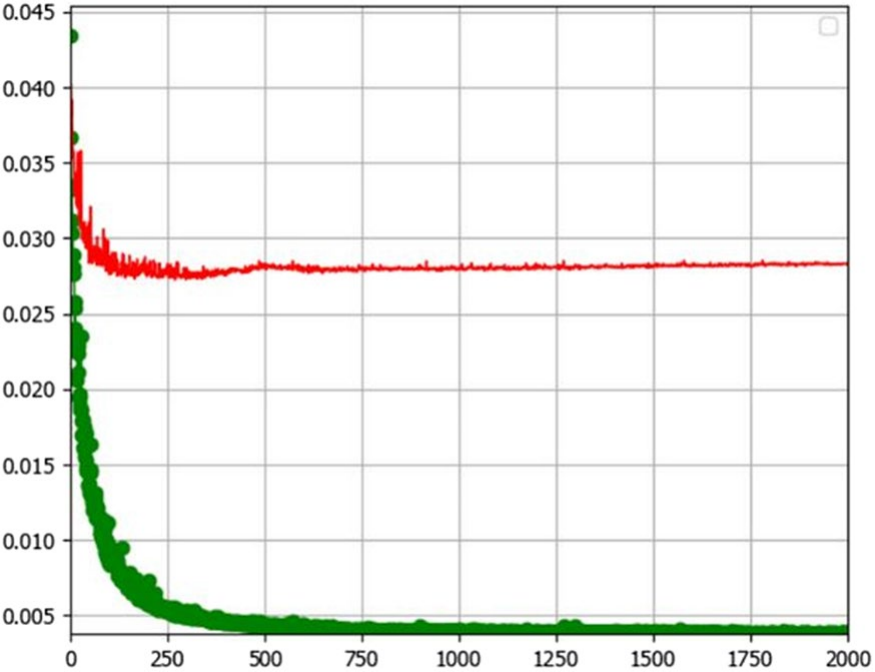
Learning curve for the 2020 CA ANN-MLP model indicating training loss

Figure 6 shows the learning curve for the calibrated model used to predict LULC in 2020. The generalization gap between the training curve (displayed in green) and validation curve (displayed in red) is too wide. This usually happens when the training data is overfit (Ding, 2021). Clearly, a decrease in the training curve can be observed. The training quality decreased as the learning experience (number of iterations) increased. Consequently, the generalization gap widened, creating a “U-shape” training curve. This means that the model was overfitted, so it could not generalize new data well enough.

## Discussion

### Supervised LULC classification

Overall, the classification results reveal urban, bare land, and cropland expansions and a general decrease in water and vegetated areas. Over the 30-year period, built-up area tripled (209%), and bare land and cropland increased both by 10%. In contrast, forest, herbaceous, waterbody area, and shrubland decreased by 30%, 4%, 20%, and 20%, respectively. Thus, the past and current temporal patterns of LULCC across southern Malawi can be summarized as follows: built-up, bare land, and cropland are increasing while forest, herbaceous, water, and shrubland are decreasing. Additionally, these findings reveal that southern Malawi is dominantly an agro-mosaic landscape with expanding urban areas and bare land, and disturbed and declining primary forests, shrublands, grasslands, and water resources.

Clearly, there is significant LULC dynamism across the region (Fig. 4). Except for cropland, all LULC types showed substantial changes in their spatial distribution over time, particularly forest, herbaceous, and shrubland cover. This reveals that, in the study area, vegetation areas often undergo transition. This result is consistent with the findings from sub-district level and district-level studies across the region (Bone et al., 2017; Mawenda et al., 2020; Minde et al., 2001).

Counter to what the authors expected, and perhaps in reflection of the moderate performance of the SVM model and differences in quality of the Landsat 5 and 8 sensors, findings from the 2000 and 2020 LULC mapping contradict the hypothesized situation and the traditional view that the increase in built-up area and cropland will reduce forest land, shrubland, and waterbody area while increasing bare land. As it turned out, the LULCC in 2000 and 2020 was striking and progressed to different outcomes, graphically. In 2000, forest land, shrubland, and waterbody area increased. Why is this the case? The increase in herbaceous and waterbody area could be associated with the extreme heavy rainfall in 2000/1 caused by Tropical Cyclone Astride (Clay et al., 2003). Similarly, forest and shrubland increased in the recent years (2010–2020 period), indicating the positive impacts of forest and land management policies. The region has two national parks (Lengwe and Liwonde), two wildlife reserves (Majete and Mwabvi), forest reserves (Machinga, Mangochi, Mulanje, Zomba, Michiru, and many others), and timber plantations found in Thyolo. This means that conservation and restoration efforts are likely to cause an increase in vegetation cover. Since 1990, these habitats have undergone significant degradation, mainly because of encroachment and deforestation (Bone et al., 2017; Kalipeni, 1992; Mawenda et al., 2020; Zulu, 2010). However, from 2015 onwards, the conservation areas have been undergoing restoration (Bone et al., 2017; Kpienbaareh et al., 2022).

This then reasonably reveals that the anthropogenic activities and climate across this region cause noticeable LULC transitions at a landscape level. Thus, this study through land cover mapping demonstrates that proximity to major roads and villages, population density, poverty, alongside temperature, precipitation, slope, and elevation provide a reasonable explanation to the occurrence of open habitats or nonforested areas at decadal intervals.

### Evaluating the effectiveness of the LULC classification model

What is evident from the classification results is that the optimised SVM classifier has proved to be not only useful in classifying heterogenous land cover, but also land cover of similar spectral signature (e.g., cropland and shrubland). In summary, a highest overall accuracy of 94% was observed in the 2020 Landsat 8 OLI. For the Landsat 5 TM, the highest overall accuracy was observed in the 1990 image (91%), followed by the 2020 image (89%), and lastly, the 2000 image (85%). The classifier showed high accuracy in discriminating bare land, followed by forest. However, pixels in the built-up, herbaceous, water, cropland, and shrubland were often misclassified. This means that the classifier yielded moderate accuracy for these four LULC classes across the study area. It could be argued that during the dry season, low density of green as a result of dry conditions in the shrubland, alongside bush fires, exposes the soil, causing similar spectral signatures between the shrubland and cleared cropland/cleared land. The classifier also frequently misclassified water pixels as herbaceous. In the study area, wetlands are dominated by different types of emergent vegetation (partly submerged plants) and water, making these classes difficult to differentiate. This was evident in areas with standing water and floodplains. However, the reason for the misclassification of water with shrubland is not clear. This is an important result for future research.

These findings broadly corroborate the findings of Kpienbaareh et al. (2022) and Palamuleni et al. (2007) who demonstrated that the close association of LULC classes often leads to mixed pixels, particularly in savanna landscapes where habitats are spatially clustered and scattered and have gradual boundaries. Thus, in addition to Clinton et al. (2010), who reported that classification inaccuracy is a resultant of poor classifier and/or poor segmentation, this study suggests that classification accuracy is also affected by spatial patterns in habitat (LULC) distribution.

### LULC simulation

The simulation reveals an intricate LULCC dynamic system, broadly a resultant of the non-linear interplay of land use and climate. Using Landsat 5 and 8 images, CA ANN-MLP model, and evidential reasoning, it has been shown that significant spatiotemporal changes in LULC occurred under development, conservation, and long-term variability of climate. Thus, we deduce that southern Malawi has a strong record of LULC dynamism shaped inclusively by land-use (agriculture, urbanization), topography and climate. Despite the interplay not being apparent, the long-term temporal LULC changes are consistent with built-up area and cropland expansions and climate.

Of note, cropland (76.2%) was the main LULC in the predicted map, followed by shrubland (9.6%). So in the actual map—cropland and shrubland areas were 72.2% and 10.7%, respectively (Table 5). Similarly, in both maps, bare land was the least land cover type, 0.9% in the actual map and 1.0% in the predicted map. Overall, the differences in area of LULC classes between the actual and simulated are minimal, attesting to the similarity between the two maps. This then means that proximity to major roads and villages, population density, poverty, alongside temperature, precipitation, slope, and elevation across the study area do directly and indirectly (1) cause significant perturbations in land use-land cover, in general, and (2) result in expansion and dominance of cropland, expansion and persistence of bare land, decline in water area, expansion of built-up, decline in vegetated areas in some areas, and persistence in vegetation in others, specifically.

### Evaluating effectiveness of the LULC simulation model

The simulated map showed good agreement with the reference map (Fig. 5). This indicates that the climate, topographic, and socio-economic predictor variables provided acceptable LULC simulation results. However, the hyperparameters used in this prediction model, namely, learning rate, momentum, and number of hidden layers, did not converge towards the least minimum error (i.e., best fit). As evidenced in the learning curve (Fig. 6), the ability of the calibrated models to learn is decreasing with experience. Clearly, this indicates overfitting in the model. Overfitting means that the model has learned the data, statistical noise, and errors too well and thereby is less capable to generalize to new data (Igiri et al., 2015; Sohil et al., 2022).

This, then, suggests that the prediction model also learned LULC patterns caused by random processes rather than by the explanatory variables. This problem is likely to be related to the main drawback of the least mean squares (LMS) algorithm in the ANN-MLP, which is used to minimize the error in the network (Collobert & Bengio, 2004). As with high-order polynomials, the LMS suffers from “ill-condition” problem, where a small change in the input results in a significant change in the output (Deng et al., 2009). In principle, the overfitting can be minimized by reducing the learning rate and/or number of the hidden layers. However, the ANN is stochastic, and the LMS is sensitive to the propagation of its input, making it “very hard (if not impossible) to choose a learning rate that guarantees stability of the algorithm” (Haykin, 2002, para.2).

The wide gap between the training and validation curves indicated that the model was trained for too long, and the training dataset is unrepresentative. Possible explanations for this are the following: (1) the number of iterations was set too high (2000 iterations), causing the model to learn for too long, and (2) the complex and multivariate nature of the input variables used here is making the model draw unrepresentative samples from one dataset, in comparison to another dataset. Consequently, the identification of the intricate patterns by the model proved difficult.

## Conclusion

Using Landsat 5 and 8 images, SVM classifier, CA ANN-MLP model, and evidential reasoning, it has been shown that significant spatiotemporal changes in LULC occurred under development, conservation, and long-term variability of climate. Thus, we conclude that southern Malawi has a strong record of LULC dynamism shaped inclusively by land use (agriculture, urbanization), topography, and climate. Despite the interplay not being apparent, the long-term temporal LULC changes are consistent with built-up area and cropland expansions under business-as-usual climate change. Overall, the LULCC trend across southern Malawi presents a threat to the biodiversity across the region. The long-term vegetation loss does not bode well with the spatial distribution of natural habitats. Thus, the LULC trend merits stepped-up conservation and restoration efforts.

This study points out the need for further research to (1) investigate the effect of band combinations on the SVM classifier accuracy, (2) elucidate the influence of the above explanatory variables on the LULC transition, (3) explicate the effect of urban and cropland expansion on spatial heterogeneity of forest and shrubland habitats and influence of fire regimes and surficial geology on long-term vegetation distribution, and (4) experiment parameter optimization using the stochastic optimization algorithms outside the MOLUSCE framework. This is motivation to develop a QGIS plugin for automatically determining optimal hyperparameters and variable combinations.

In conclusion, greater understanding of LULCC in southern Malawi will not only require coarse-grained stochastic climatic models or advanced hybrid socio-economic models, but also using fine-resolution data or less explored ecological predictors (or both).

## Data Availability

The data that support the findings of this study are available from the corresponding author, (CN), upon reasonable request.

## Supplemetary information

ESM 1 (DOCX 688 kb)
