# Supplementary material for: Monitoring and simulating landscape changes: how do long-term changes in land use and long-term average climate affect regional biophysical conditions in southern Malawi?
Source: Environ Monit Assess. 2023 Sep 26;195(10):1247. doi: 10.1007/s10661-023-11783-9 (PMC10522741; doi:10.1007/s10661-023-11783-9)
Supplement: ESM 1 — (DOCX 688 kb) [file 10661_2023_11783_MOESM1_ESM.docx]

# Appendix

**A**


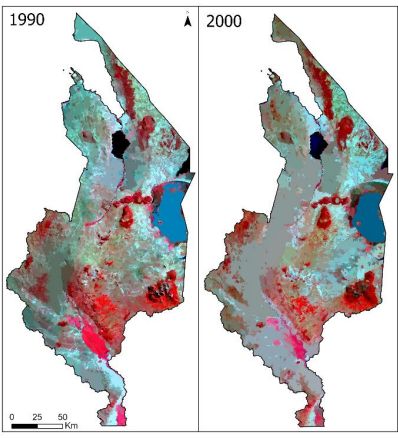


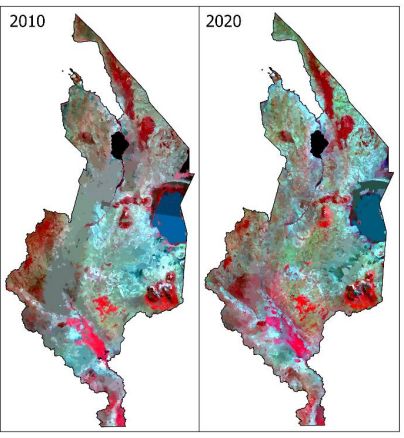


The segmented Landsat 5 TM (1990, 2000 and 2010) and 8 OLI (2020) false colour images used in the LULC classification. (Source: Own representation).

**B**

(a)


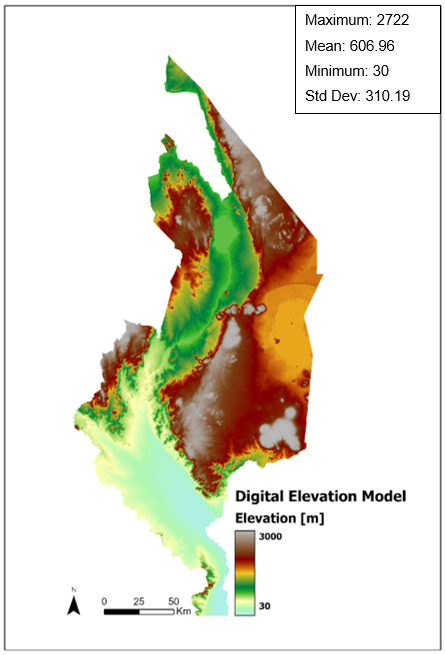


(b)


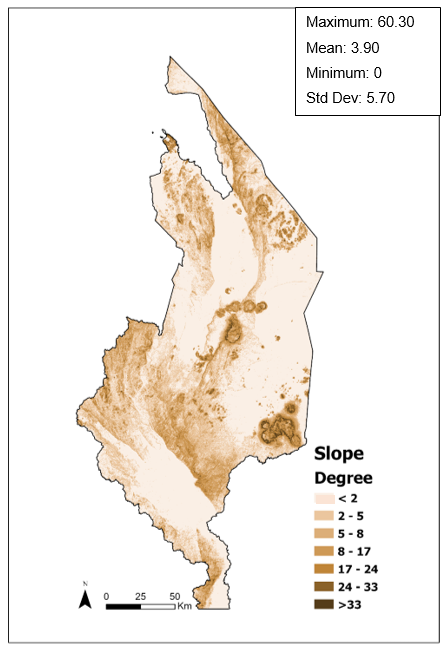


(c)


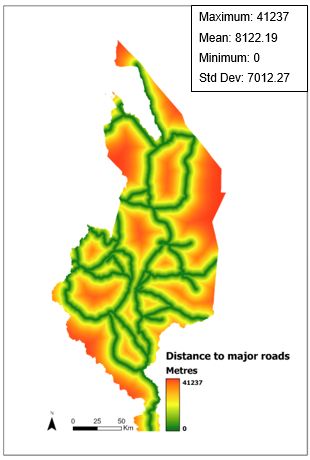


(a) The elevation gradient across southern Malawi is strong. In contrast to the eastern central areas of the region, the southernmost areas are low lying. In general, the topography sinks from the north-east to the south-central. (b) Variation in the slope across southern Malawi. The slope is not uniform. (c) Euclidean distance to major roads. (Source: Own representation).

**C**

(a)

**
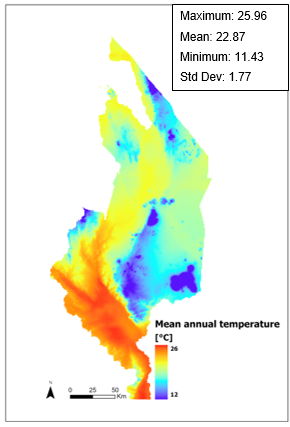
**

(b)


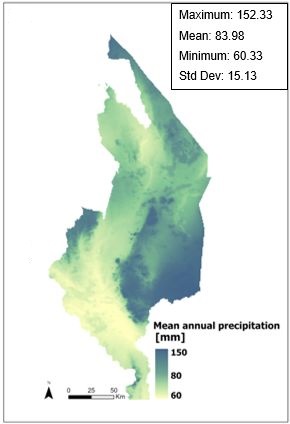


Past (1970-2000) annual mean temperature (a) and precipitation (b). (Data source: WorldClim, 2022). (Source: Own representation).

**D**

(a)


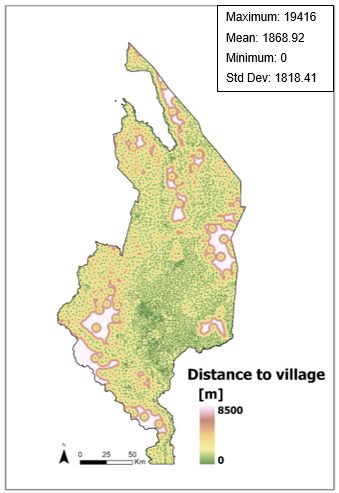


(b)


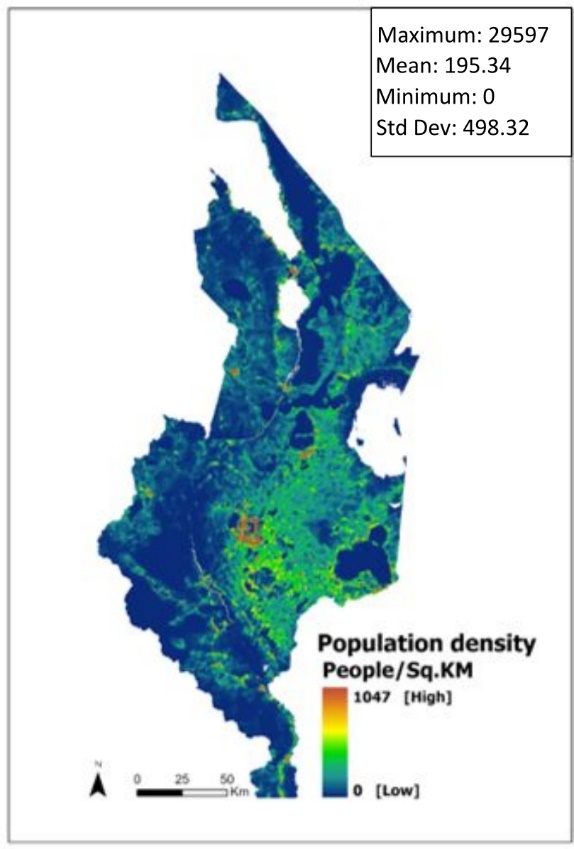


(c)


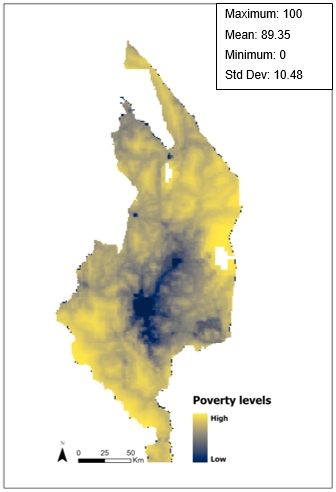


(a) Euclidean distance to villages. (b) Population density. The population is mostly concentrated in the central eastern areas. This means that these areas have the highest population density, indicating that they are urban centres. A broader pattern emerges from the evidence presented thus far: human footprint across the region is concentrated around the central-eastern area. (c) Poverty levels. A spatial pattern emerges when the poverty map is compared with the village density and population density maps: population centres have low levels of poverty, and vice versa. (Source: Own representation).
